# Joint effects of female preference intensity and frequency‐dependent predation on the polymorphism maintenance in aposematic sexual traits

**DOI:** 10.1002/ece3.9356

**Published:** 2022-10-11

**Authors:** Aditya Ponkshe, John A. Endler

**Affiliations:** ^1^ Centre for Integrative Ecology, School of Life & Environmental Sciences Deakin University Waurn Ponds Victoria Australia; ^2^ MINT Lab, Edificio Luis Vives, Campus Espinardo University of Murcia Murcia Spain

**Keywords:** aposematism, Fisher process, frequency‐dependent predation, mate choice, polymorphism maintenance, warning signals

## Abstract

Maintenance of variation in aposematic traits within and among populations is paradoxical because aposematic species are normally under positive frequency‐dependent predation (PFD), which is expected to erode variation. Aposematic traits can evolve in an ecological context where aposematic traits are simultaneously under mate choice. Here, we examine how the mate preference intensity affects the permissiveness of polymorphism in sexually selected aposematic traits under different PFD regimes. We use the haploid version of the classical sexual selection model and show that strong mate preferences can substantially increase the permissiveness of polymorphism in aposematic traits under different PFD regimes. The Fisher process can interact with PFD, and their interaction can promote the maintenance of polymorphism within populations when mate preferences are strong. We show that the same selective conditions that promote the maintenance of polymorphism within populations reduce the likelihood of divergence in aposematic traits among populations.

## Graphical abstract

We examine how the mate preference intensity affects the polymorphism maintenance in aposematic traits affected by positive frequency‐dependent predation (PFD). We use the haploid version of the classical sexual selection model and show that the Fisher process interact with PFD and maintain polymorphism in aposematic traits within populations when mate preferences are strong. We show that selective conditions that promote the maintenance of polymorphism within populations reduce the likelihood of divergence in aposematic traits among populations.

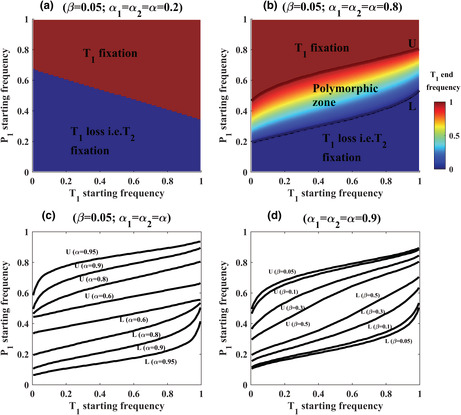

## 1 INTRODUCTION

Classical theory suggests that aposematic traits should have low variance (Endler, 1988; Endler & Mappes, 2004), yet many species show variation in these traits within and between populations in a variety of taxa [beetles (Borer et al., 2010; Brakefield, 1985; O'donald & Majerus, 1984), moths (Gordon et al., 2015; Nokelainen et al., 2012) and frogs (Rojas & Endler, 2013; Siddiqi et al., 2004)]. Maintenance of variation in aposematic traits within and among populations becomes paradoxical because aposematic traits are normally under positive frequency‐dependent natural selection due to frequency‐dependent predation (Borer et al., 2010; Briolat et al., 2019; Chouteau et al., 2016; Gordon et al., 2015). In PFD, fitness increases with the phenotype's frequency or density (Endler, 1988; Endler & Mappes, 2004; Mallet & Joron, 1999; Noonan & Comeault, 2009). If a trait's fitness is positive frequency‐dependent, then purifying selection is expected to erode variation and populations typically evolve to one of the alternative stable states depending on where they start in the allele frequency space (Endler & Mappes, 2004; Lehtonen & Kokko, 2012).

Aposematic traits can evolve in an ecological context where traits are simultaneously under mate choice. Aposematic species can be under PFD and additionally can show variation in mate preferences based on warning color components (Gordon et al., 2015; Maan & Cummings, 2008; Summers et al., 1999). Variable Strawberry poison‐dart frog (*Oophaga pumilio*) is an iconic example of such interaction where both males and females express warning colors and females use visual cues during mate choice (Maan & Cummings, 2008; Siddiqi et al., 2004; Summers et al., 1999) and prefer matching males over non‐matching males (Reynolds & Fitzpatrick, 2007). Recent developments in sexual selection theory suggest that on their own, mate preferences can promote the maintenance of sexual trait diversity (M'Gonigle et al., 2012; Ponkshe & Endler, 2018; Servedio & Bürger, 2014). However, how mate preferences contribute to the maintenance of polymorphism in PFD‐affected sexual traits is largely neglected. Here, we examine how mate preference intensity affects the permissiveness of sexual trait polymorphism in PFD‐affected environments. Permissiveness of polymorphism can be described as the capacity of environments to allow the polymorphism to be maintained in variable conditions (Ponkshe & Endler, 2018). We use Kirkpatrick's haploid version of the classical model of sexual selection (Kirkpatrick, 1982) as a foundation to address this question.

Kirkpatrick's classical model of sexual selection is based on the Fisher process, which is a null process of trait‐preference co‐evolution and remains at the core of sexual selection (Prum, 2010), but also see Kovaka (Kovaka, 2020) for further discussion on the null model of sexual selection. Kirkpatrick's original model and other genetic models expanding it further normally discuss implications of the Fisher process in the context of trait‐preference exaggeration and speciation (Fuller et al., 2005; Houde, 1993; Kirkpatrick, 1982; Kuijper et al., 2012; Lande, 1981; Prum, 2010; Uyeda et al., 2009). Instead, here we use Kirkpatrick's model as a foundation and examine how the Fisher process interacts with PFD and under which conditions their interaction promotes versus constrains the maintenance of polymorphism in aposematic traits within populations. Additionally, we identify conditions that promote versus reduce the likelihood of divergence in aposematic traits among populations.

Population genetic models expanding the classical model of sexual selection generally include directional natural selection on traits and/or preferences (Bulmer, 1989; Kuijper et al., 2012; Seger, 1985; Seger & Trivers, 1986; Takahasi, 1997). In almost all aposematic species, both males and females express warning signals, and both are affected by PFD independent of mate choice. Consequently, we consider a scenario where both males and females are affected by PFD.

## 2 MODEL AND RESULTS

Consider a haploid population showing polymorphism in sexual traits and mating preferences. Assume that PFD‐affected traits are expressed in both males and females and are controlled by locus T, whereas unlinked locus P controls mate preferences in females. Let locus T have two alleles T_1_ and T_2,_ which correspond to different sexual traits, and locus P have two alleles P_1_ and P_2,_ which correspond to different mate preferences (Kirkpatrick, 1982; Ponkshe & Endler, 2018). We assume both males and females are affected by ecologically driven positive frequency‐dependence (PFD).

Let *m*_*1*_, *m*_*2*_, *m*_*3*,_ and *m*_*4*_ represent the starting frequencies of T_1_P_1_, T_1_P_2_, T_2_P_1,_ and T_2_P_2_ zygotes in males and *f*_*1*_, *f*_*2*_, *f*_*3*,_ and *f*_*4*_ be their starting frequencies in females. Let *β* be the strength of PFD. Consequently, male genotype fitness measures are

$$\;W_{T_{1}P_{1}}=1+\beta \left(m_{1}+m_{2}\right);W_{T_{1}P_{2}}=1+\beta \left(m_{1}+m_{2}\right);$$

$$W_{T_{2}P_{1}}=1+\beta \left(m_{3}+m_{4}\right);W_{T_{2}P_{2}}=1+\beta \left(m_{3}+m_{4}\right)$$

Let PFD on sexual traits occur before mating; this alters the frequencies of males available for mating. Male genotype frequencies available for selective mating after PFD are

$$\begin{array}{c} m_{1}^{\prime }=\frac{m_{1}\left(1+\beta \left(m_{1}+m_{2}\right)\right)}{{\bar{W}}_{\text{males}}};m_{2}^{\prime }=\frac{m_{2}\left(1+\beta \left(m_{1}+m_{2}\right)\right)}{\;{\bar{W}}_{\text{males}}}; \end{array}$$

$$\begin{array}{c} m_{3}^{\prime }=\frac{m_{3}\left(1+\beta \left(m_{3}+m_{4}\right)\right)}{{\bar{W}}_{\text{males}}};m_{4}^{\prime }=\frac{m_{4}\left(1+\beta \left(m_{3}+m_{4}\right)\right)}{{\bar{W}}_{\text{males}}} \end{array}$$

where,

$$\begin{array}{cc} {\bar{W}}_{\text{males}}= & [m_{1}\left(1+\beta \left(m_{1}+m_{2}\right)\right)+m_{2}\left(1+\beta \left(m_{1}+m_{2}\right)\right)\\ & +m_{3}\left(1+\beta \left(m_{3}+m_{4}\right)\right)+m_{4}\left(1+\beta \left(m_{3}+m_{4}\right)\right)]. \end{array}$$

Since PFD is on T in both males and females, female genotype fitness measures are

$$W_{T_{1}P_{1}}=1+\beta \left(f_{1}+f_{2}\right);W_{T_{1}P_{2}}=1+\beta \left(f_{1}+f_{2}\right);$$

$$W_{T_{2}P_{1}}=1+\beta \left(f_{3}+f_{4}\right);W_{T_{2}P_{2}}=1+\beta \left(f_{3}+f_{4}\right)$$

Frequencies of female genotypes available for mating after PFD are

$$\begin{array}{c} \;f_{1}^{\prime }=\frac{f_{1}\left(1+\beta \left(f_{1}+f_{2}\right)\right)}{{\bar{W}}_{\text{females}}};f_{2}^{\prime }=\frac{f_{2}\left(1+\beta \left(f_{1}+f_{2}\right)\right)}{\;{\bar{W}}_{\text{females}}}; \end{array}$$

$$\begin{array}{c} f_{3}^{\prime }=\frac{f_{3}\left(1+\beta \left(f_{3}+f_{4}\right)\right)}{{\bar{W}}_{\text{females}}};f_{4}^{\prime }=\frac{f_{4}\left(1+\beta \left(f_{3}+f_{4}\right)\right)}{{\bar{W}}_{\text{females}}} \end{array}$$

where,

$$\begin{array}{cc} {\bar{W}}_{\text{females}}= & [f_{1}\left(1+\beta \left(f_{1}+f_{2}\right)\right)+f_{2}\left(1+\beta \left(f_{1}+f_{2}\right)\right)\\ & +f_{3}\left(1+\beta \left(f_{3}+f_{4}\right)\right)+f_{4}\left(1+\beta \left(f_{3}+f_{4}\right)\right)]. \end{array}$$

Let P_1_ females prefer T_1_ males with the relative preference 1 and let her preference for T_2_ males be 1‐*α*_*1*_. Similarly, let P_2_ prefer T_2_ males with the relative preference 1 and let her preference for T_1_ males be 1‐*α*_*2*_ *. α*_*1*_ and *α*_*2*_ are mate choice coefficients where *α*_*1*_ = *α*_*2*_ = 0 means no choice with respect to male traits and *α*_*1*_ = *α*_*2*_ = 1 means both females only mate with their preferred males. Next generation zygote frequencies were obtained by substituting male and female haplotype frequencies into the recursion equations

(1) $$T_{1}{P_{1}}_{\left(t+1\right)}={f_{1}}^{\prime }\left[\frac{m_{1}^{\prime }}{z_{1}}+\frac{m_{2}^{\prime }}{2z_{1}}+\frac{m_{3}^{\prime }\left(1-\alpha_{1}\;\right)}{2z_{1}}+\frac{m_{4}^{\prime }\left(1-\alpha_{2}\;\right)}{4z_{1}}\right]+{f_{3}}^{\prime }\left[\frac{m_{1}^{\prime }}{2z_{1}}+\frac{m_{2}^{\prime }}{4z_{1}}\right]+{f_{2}}^{\prime }\left[\frac{m_{1}^{\prime }\left(1-\alpha_{2}\;\right)}{2z_{2}}+\frac{m_{3}^{\prime }}{4z_{2}}\right]+{f_{4}}^{\prime }\left[\frac{m_{1}^{\prime }\left(1-\alpha_{2}\;\right)}{4z_{2}}\right]$$

(2) $$T_{1}{P_{2}}_{\left(t+1\right)}={f_{2}}^{\prime }\left[\frac{m_{1}^{\prime }\left(1-\alpha_{2}\right)}{2z_{2}}+\frac{m_{2}^{\prime }\left(1-\alpha_{2}\;\right)}{z_{2}}+\frac{m_{3}^{\prime }}{4z_{2}}+\frac{m_{4}^{\prime }}{2z_{2}}\right]+{f_{4}}^{\prime }\left[\frac{m_{1}^{\prime }\left(1-\alpha_{2}\;\right)}{4z_{2}}+\frac{m_{2}^{\prime }\left(1-\alpha_{2}\;\right)}{2z_{2}}\right]+{f_{1}}^{\prime }\left[\frac{m_{2}^{\prime }}{2z_{1}}+\frac{m_{4}^{\prime }\left(1-\alpha_{1}\;\right)}{4z_{1}}\right]+{f_{3}}^{\prime }\left[\frac{m_{2}^{\prime }}{4z_{1}}\right]$$

(3) $$T_{2}{P_{1}}_{\left(t+1\right)}={f_{1}}^{\prime }\left[\frac{m_{3}^{\prime }\left(1-\alpha_{1}\right)}{2z_{1}}+\frac{m_{4}^{\prime }\left(1-\alpha_{1}\right)}{4z_{1}}\right]+{f_{3}}^{\prime }\left[\frac{m_{1}^{\prime }}{2z_{1}}+\frac{m_{2}^{\prime }}{4z_{1}}+\frac{m_{3}^{\prime }\left(1-\alpha_{1}\right)}{z_{1}}+\frac{m_{4}^{\prime }\left(1-\alpha_{1}\right)}{2z_{1}}\right]+{f_{2}}^{\prime }\left[\frac{m_{3}^{\prime }}{4z_{2}}\right]+{f_{4}}^{\prime }\left[\frac{m_{1}^{\prime }\left(1-\alpha_{2}\right)}{4z_{2}}+\frac{m_{3}^{\prime }}{2z_{2}}\right]$$

(4) $$T_{2}{P_{2}}_{\left(t+1\right)}={f_{2}}^{\prime }\left[\frac{m_{3}^{\prime }}{4z_{2}}+\frac{m_{4}^{\prime }}{2z_{2}}\right]+{f_{4}}^{\prime }\left[\frac{m_{1}^{\prime }\left(1-\alpha_{2}\right)}{4z_{2}}+\frac{m_{2}^{\prime }\left(1-\alpha_{2}\right)}{2z_{2}}+\frac{m_{3}^{\prime }}{2z_{2}}+\frac{m_{4}^{\prime }}{z_{2}}\right]+{f_{1}}^{\prime }\left[\frac{m_{4}^{\prime }\left(1-\alpha_{1}\right)}{4z_{1}}\right]+{f_{3}}^{\prime }\left[\frac{m_{2}^{\prime }}{4z_{1}}+\frac{m_{4}^{\prime }\left(1-\alpha_{1}\right)}{2z_{1}}\right]$$

here,

$$\begin{array}{c} z_{1}={m_{1}}^{\prime }+{m_{2}}^{\prime }+\left(1-\alpha_{1}\right)\left({m_{3}}^{\prime }+{m_{4}}^{\prime }\right);\\ z_{2}=\left(1-\alpha_{2}\right)\left({m_{1}}^{\prime }+{m_{2}}^{\prime }\right)+{m_{3}}^{\prime }+{m_{4}}^{\prime } \end{array}$$

We examined the model behavior in MATLAB 2021a by numerically computing equilibrium T_1_ frequencies after 5000 generation which we found sufficient time for the populations to reach equilibrium. Populations were considered at equilibrium when there was no difference in allele frequencies among consecutive generations. We computed equilibrium T_1_ frequencies for the entire T_1_‐P_1_ space. Note that for every combination of *β*, *α*_*1*,_ and *α*_*2*,_ the starting T_1_ and P_1_ frequencies were distributed among all four genotypes.

**FIGURE 1:**
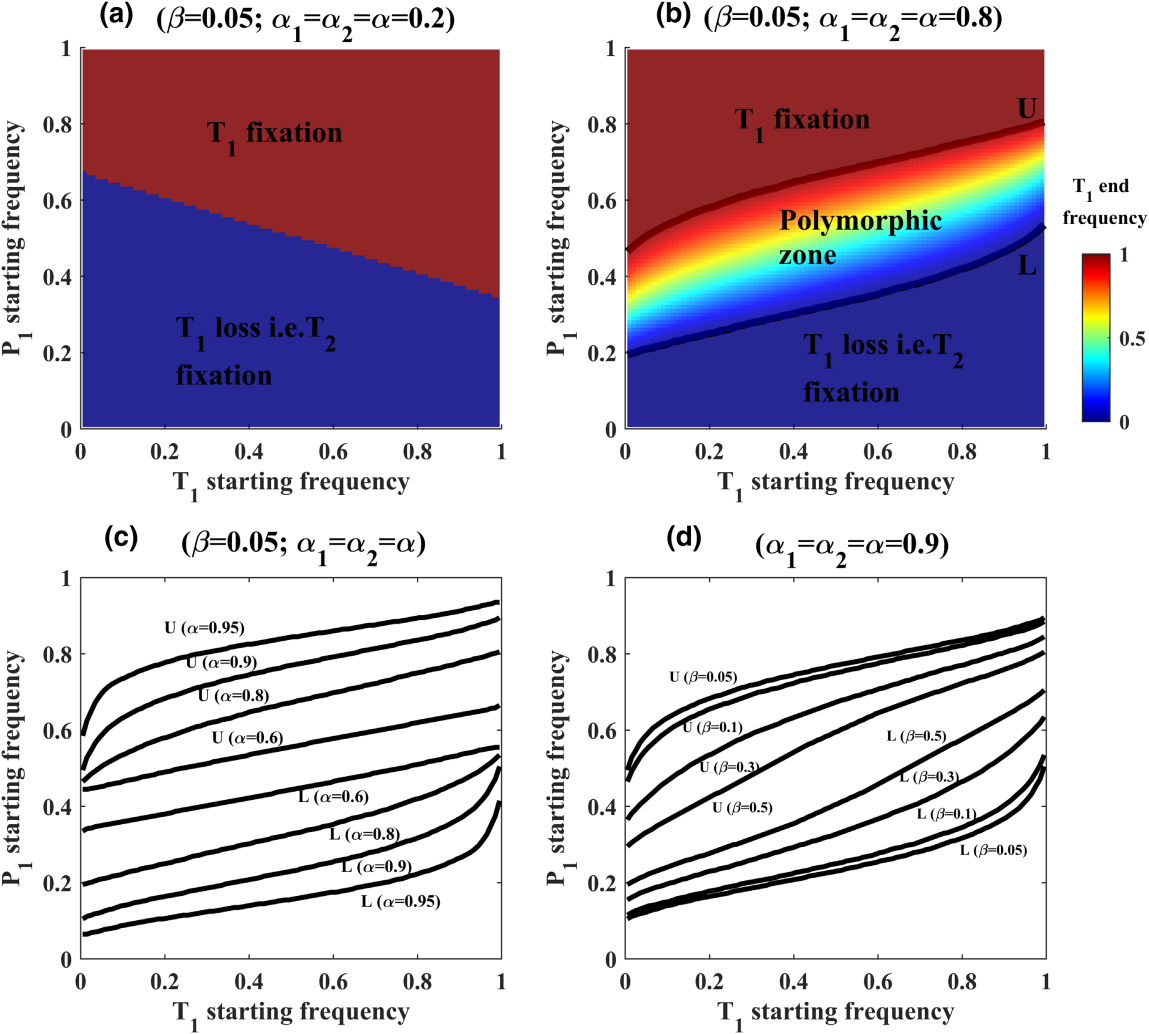
Effects of mate choice and PFD parameters (*α*_*1*_, *α*_*2*,_ and *β*) on polymorphic zones. (a) Phase map showing attraction basin of T_1_ fixation and T_1_ loss, that is, T_2_ fixation when mate preference (*α*) and PFD are weak (*α*_*1*_ = *α*_*2*_ = *α* = 0.2; *β* = 0.05). Note that the polymorphic zone remains absent in this case. (b) Phase map showing the polymorphic zone, delimited by two thresholds, U and L (thick black curves), when PFD strength (*β*) is weak (*β* = 0.05) and *α* is strong (*α*_*1*_ = *α*_*2*_ = *α* = 0.8). (c) Changes in U and L as a function of mate preference strength under weak PFD (*β* = 0.05). (d) Changes in U and L as a function of *β* when mate preferences are symmetric and strong (*α*_*1*_ = *α*_*2*_ = *α* = 0.9).

For a combination of PFD strength (*β*) and mate preferences (*α*_*1*_ and *α*_*2*_), joint trait‐preference starting frequencies that will maintain polymorphism in T at equilibrium form a zone in the trait‐preference frequency space (Figure 1b). Following Ponkshe and Endler (Ponkshe & Endler, 2018, 2019), we refer this zone as polymorphic zone. The polymorphic zone has two distinct boundaries. We call them as the upper (U) and lower (L) boundaries based on their intersection on the axis of P_1_ starting frequencies (Figure 1b). The three zones defined by U and L produce very different evolutionary outcomes. Populations remain polymorphic if they fall inside the polymorphic zone. If populations fall above U or below L, populations can either fix or lose the same trait allele. However, different sexual trait alleles will go to fixation if populations happen to fall on the opposite sides of the polymorphic zone. In the narrow polymorphic zone, polymorphic populations are more likely to be sensitive to perturbations in allele frequencies than in the broad zone because populations are more likely to cross zone boundaries by a transitory change in allele frequencies when the zone is narrow than when it is broad.

We followed Ponkshe and Endler (Ponkshe & Endler, 2018, 2019) to identify the polymorphic zone and boundaries. First, for a given PFD (*β*) and *α*_*1*_‐*α*_*2*_ combination, we computed the equilibrium T_1_ frequency for all possible combinations of T_1_‐P_1_ starting frequencies. The polymorphic zone includes all T_1_‐P_1_ starting frequencies that produce equilibrium T_1_ frequency between 0.001 and 0.999 (0.001 < T_1 [equilibrium frequency]_ < 0.999). To compute U, we identified a threshold P_1_ for all T_1_ such that any change in P_1_ above U will give T_1_ fixation, that is, T_1 (equilibrium frequency)_ > 0.999 (see U in Figure 1b). Similarly, to compute L, we identified a threshold P_1_ for all T_1_ such that any change in the starting frequency of P_1_ below L will result in T_1_ loss, that is, T_1 (equilibrium frequency)_ < 0.001 (see L in Figure 1b).

We will illustrate model results under weak (*β* = 0.05), moderate (*β* = 0.1), and strong (*β* = 0.5) PFD regimes. *β* reported in a natural population of aposematic leaf beetles (*Oreina gloriosa*) is 0.13 (selection corresponds to 13% against foreign morph relative to locally common morph) (Borer et al., 2010). In other Mullerian mimicry systems, estimates of *β* range from 0.22 to 0.6 (Benson, 1972; Kapan, 2001; Mallet & Barton, 1989).

### 2.1 Joint effects of PFD and mate preferences on the polymorphic zone

**FIGURE 2:**
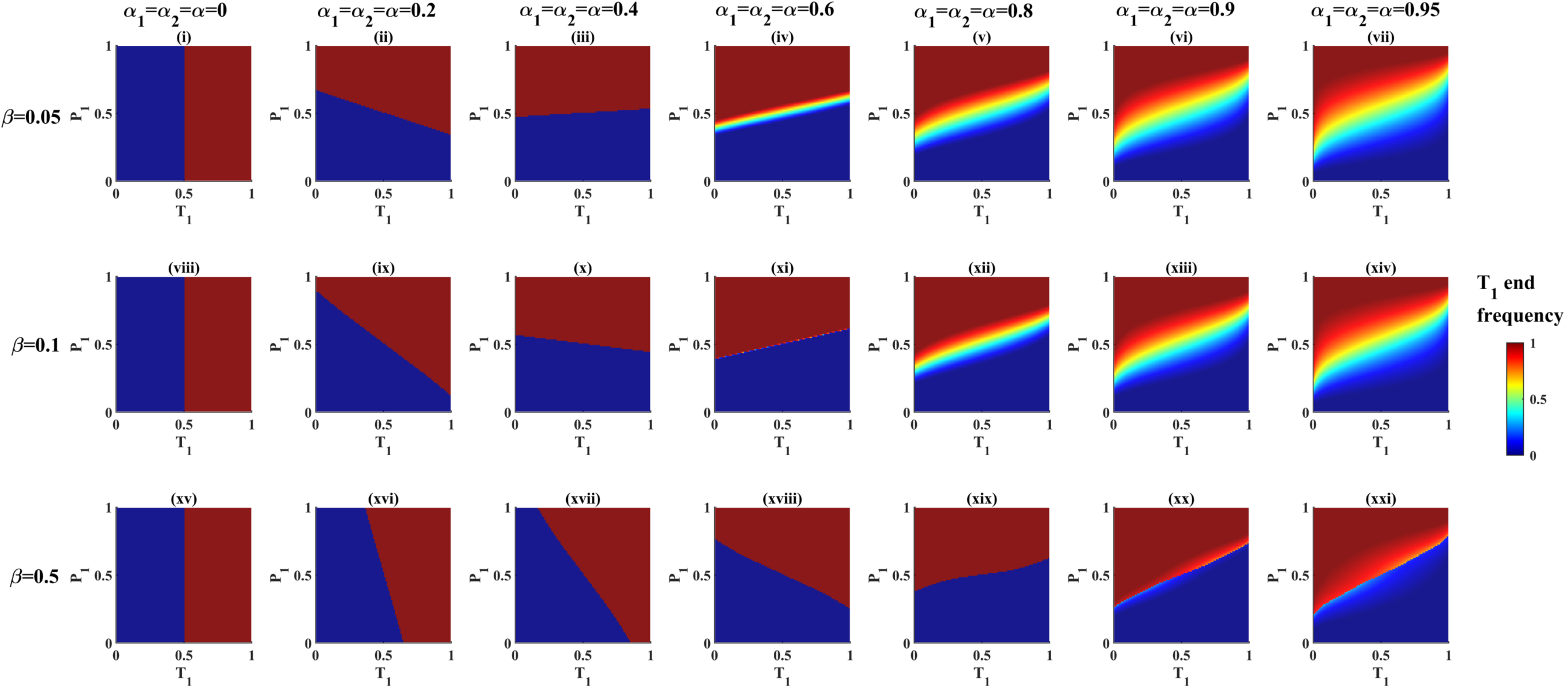
Consequences of interaction between PFD and mate preferences when mate preferences are symmetric. (i to vii) Phase maps showing evolutionary outcomes as a function of mate preference strength when PFD is weak (*β* = 0.05). (viii to xiv) Phase maps showing evolutionary outcomes as a function of mate preference strength when PFD is moderate (*β* = 0.1). (xv to xxi) Phase maps showing evolutionary outcomes as a function of mate preference strength when PFD is strong (*β* = 0.5). Note that in all panels, axes labels P_1_ and T_1_ refer to P_1_ starting frequency and T_1_ starting frequency respectively.

Different combinations of *α*_*1*_, *α*_*2*,_ and *β* alter the position, shape, and size of the polymorphic zone. Joint trait‐preference starting frequencies form the polymorphic zone when mate preferences are strong. The polymorphic zone and zone boundaries (U and L) are only present when mate preferences are strong, that is, *α*_*1*_ = *α*_*2*_ = *α* > 0.5 in Figure 1c. The polymorphic zone remains absent when mate preferences are weak under the weak, moderate and strong PFD regimes (Figure 2).

For a given PFD strength (*β*), more trait‐preference starting frequency combinations maintain sexual trait polymorphism as mating strength *α* increases. As a result, the polymorphic zone gradually increases in size as *α* increases. Note the systematic expansion between U and L with a gradual increase in *α* in Figure 1c when PFD is weak (*β* = 0.05). Also note the expansion of the polymorphic zone under moderate (*β* = 0.1) PFD regime in Figure 2 (compare polymorphic zones in Figure 2xii,xiii,xiv when PFD is moderate, that is, when *β* = 0.1).

The polymorphic zone remains absent when mate preferences are weak under different PFD regimes. In the absence of a polymorphic zone, starting T_1_‐P_1_ frequency space produces two evolutionary outcomes, that is, either T_1_ fixation and/or T_1_ loss, that is, T_2_ fixation (Figure 1a). the polymorphic zone appears as the system moves from weak to strong mate preferences under weak, moderate, and strong PFD regimes (Figure 2). In the absence of selective mating (*α*_*1*_ = *α*_*2*_ = *α* = 0), the threshold boundary that separates these two evolutionary outcomes (i.e., T_1_ fixation and/or T_1_ loss, i.e., T_2_ fixation) remains at T_1_ starting frequency = 0.5. In such cases, starting T_1_ frequencies determine the subsequent direction of evolution of T irrespective of starting P_1_ frequencies. If starting T_1_ frequency is > 0.5, then T_1_ is fixed, and if starting T_1_ frequency is < 0.5, then T_1_ is lost and T_2_ goes to fixation irrespective of starting female frequencies (Figure 2i,viii,xv). However, as soon as *α* exceeds 0, starting P_1_ frequencies begin to affect the subsequent direction of trait evolution (for instance, compare Figure 2i,iii and see how the shape of threshold boundary changes from vertical to almost a horizontal line).

**FIGURE 3:**
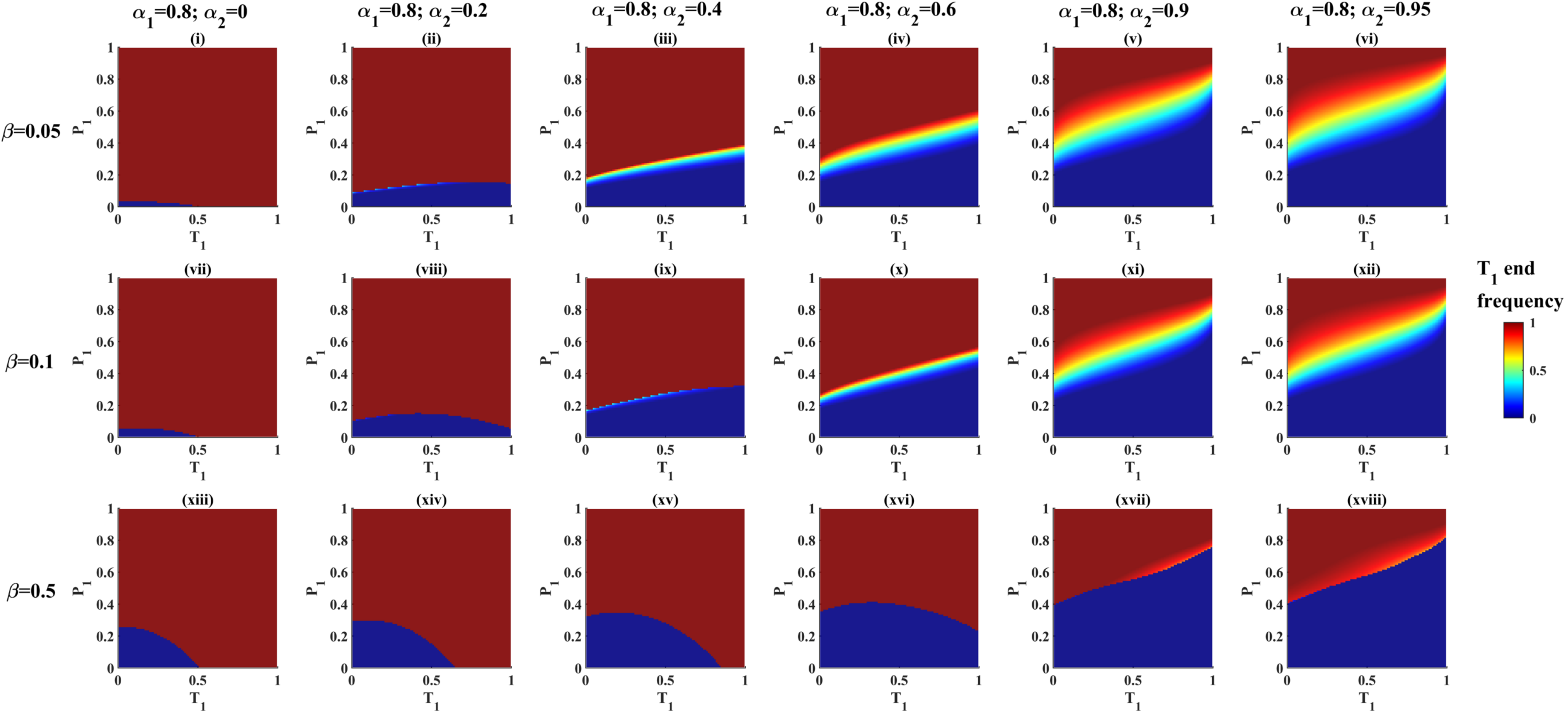
Consequences of interaction between PFD and mate preferences for unequal mate preferences: varying *α*_*2*_ and holding *α*_*1*_ constant and strong (*α*_*1*_ = 0.8). (i to vi) Phase maps showing evolutionary outcomes for varying *α*_*2*_ when PFD is weak (*β* = 0.05). (vii to xii) Phase maps showing evolutionary outcomes for varying *α*_*2*_ when PFD is moderate (*β* = 0.1). (xiii to xviii) Phase maps showing evolutionary outcomes for varying *α*_*2*_ when PFD is strong (*β* = 0.5). Note that in all panels, axes labels P_1_ and T_1_ refer to P_1_ starting frequency and T_1_ starting frequency respectively.

Figure 3 shows the relationship between the polymorphic zone and mate choice intensity (*α*) under different PFD regimes (*β*) when mate preferences are asymmetric (i.e., *α*_*1*_ ≠ *α*_*2*_). The permissiveness of polymorphism (polymorphic zone size) and its position changes disproportionately when mate preferences are asymmetric. Small differences in *α*_*1*_ and/or *α*_*2*_ among populations can produce disproportionately large differences in the permissiveness of polymorphisms in sexually selected aposematic traits.

For a constant *α*_*1*_‐*α*_*2*_ combination, polymorphic zone gradually becomes narrow and decreases in size as *β* increases. Figure 1d shows this result for strong *α* (*α*_*1*_ = *α*_*2*_ = *α* = 0.9). Note the contraction between U and L with an increase in *β* in Figure 1d. Also, Figure 2 shows this result for different combinations of *α* ranging from *α* = 0 to *α* = 0.95 under different PFD regimes.

## 3 DISCUSSION

Our study shows that strong mating preferences significantly increase the permissiveness of polymorphism in aposematic traits under a broad range of positive frequency‐dependent selection (PFD) regimes. The stronger the mate choice is, the less likely a local polymorphism is to be lost due to the chance fixation of a single morph favored by PFD. Here, we discuss the implications of the Fisher process interacting with PFD in three contexts: (1) maintaining polymorphism in PFD‐affected sexual traits within sympatric populations; (2) PFD can enhance divergence of aposematic traits in allopatric populations via Fisherian runaway; and (3) PFD may affect contact zones. Our results show that selective conditions that promote polymorphism maintenance within populations reduce the likelihood of divergence in aposematic traits among populations.

Selective mating can produce an overall negative frequency‐dependent selection (Seger, 1985) and can maintain sexual trait polymorphisms within populations when mate preferences are not under directional selection (Ponkshe & Endler, 2018). For a given PFD strength, more combinations of starting trait‐preference frequencies can maintain polymorphism in aposematic traits within populations when mate choice is strong than when it is weak. Consequently, the polymorphic zone (range of conditions favoring polymorphism) increases in size as the strength of mate choice increases. The polymorphic zone remains broad under a broad range of PFD when females show strong mate preferences for their respective male traits. For populations that sit in the middle of the polymorphic zone, relatively larger perturbations in trait‐preference frequencies are required to throw populations out of the zone and lose polymorphism when the polymorphic zone is broad than when it is narrow. Consequently, strong mating preferences make PFD‐affected polymorphic populations more resilient in the face of transitory allele frequency fluctuations.

Selective conditions that make polymorphic zones broad reduce the chances of divergence of aposematic traits. Broad polymorphic zones make it more difficult to throw populations from the polymorphic zone in opposite directions than narrow zones. Consequently, for the given range of allele frequency perturbations and for the given PFD regime, sets of polymorphic populations with strong mate preferences are less likely to diverge than sets of aposematic populations with weaker mate preferences. These results suggest that early stages of divergence in aposematic traits among populations could stall in environments with strong mate choice, and further changes in mate choice and/or PFD parameters may need to occur before populations can diverge completely.

Previous theoretical models show that strong localized PFD in tandem with dispersal can produce geographic variation in aposematic traits (Joron & Iwasa, 2005; Sherratt, 2006). Our results suggest that when the mate choice intensity is strong, differences in starting trait‐preference frequencies can potentially generate geographical variation in aposematic traits. Additionally, the small differences in *α*_*1*_ and/or *α*_*2*_ can interact with the differences in PFD parameters among populations, can cause disproportionately large differences in the size, shape, and position of polymorphic zones, and consequently can produce geographic variation in aposematic traits.

If populations are isolated by distance, then founder effects could lead to the prevalence of different morphs of warning signals in different populations (Mallet, 2010). However, it is less clear how the polymorphic zone behaves in geographic space rather than parameter space when there is migration between populations. The relationship between the permissiveness of polymorphism and gene flow is entirely unexplored in aposematic traits. On its own, the Fisher process reduces the likelihood of divergence in sexual traits among populations (Ponkshe & Endler, 2018), including in the presence of gene flow (Servedio & Bürger, 2014). If alleles coming into populations fluctuate such that directional bias changes over time, strong mate preferences coupled with low gene flow may promote the maintenance of polymorphism within populations. Such a scenario may reduce the likelihood of divergence in aposematic traits among populations.

Given that stronger mate preferences in aposematic species favor polymorphism and reduce the chances of divergence, the width of contact zones and hybrid zones should be greater for aposematic species or species pairs with stronger mate preferences. This also suggests that the differences across the contact or hybrid zone should be smaller for species with stronger mate preferences than for species with weaker mate preference. The reduction in width and magnitude should occur in both primary and secondary contact. This gives rise to the apparently counterintuitive prediction that the stronger the mate preferences the weaker the species or subspecies boundaries, if there is any gene flow. Note that this prediction only applies to aposematic species or species pairs.

In summary, the Fisher process can interact with PFD and their interaction can maintain polymorphism in aposematic traits within populations under a broad range of PFD regimes when mate preferences are strong. However, when mate preferences are strong, the interaction between the Fisher process and PFD reduces the likelihood of divergence in aposematic traits among allopatric populations.

## AUTHOR CONTRIBUTIONS

**Aditya Ponkshe:** Conceptualization (lead); formal analysis (lead); investigation (lead); methodology (lead); project administration (lead); writing – original draft (lead); writing – review and editing (lead). **John Endler:** Conceptualization (supporting); funding acquisition (supporting); supervision (lead); validation (equal); writing – review and editing (supporting).

## CONFLICT OF INTEREST

The authors have no competing interests.

## Data Availability

Data sharing not applicable to this article as no new datasets were generated or analysed during the current study
